# Mannitol-Coated Hydroxypropyl Methylcellulose as a Directly Compressible Controlled Release Excipient for Moisture-Sensitive Drugs: A Stability Perspective

**DOI:** 10.3390/ph17091167

**Published:** 2024-09-04

**Authors:** Christina Yong Xin Kang, Keat Theng Chow, Yuan Siang Lui, Antoine Salome, Baptiste Boit, Philippe Lefevre, Tze Ning Hiew, Rajeev Gokhale, Paul Wan Sia Heng

**Affiliations:** 1 Roquette Asia Pacific Pte. Ltd., 11 Biopolis Way, Helios, #05-06, Singapore 138667, Singapore; 2 GEA-NUS Pharmaceutical Processing Research Laboratory, Department of Pharmacy, National University of Singapore, 18 Science Drive 4, Singapore 117543, Singapore; paulwsheng@outlook.com; 3 Roquette Frères, 1 Rue de la Haute Loge, 62136 Lestrem, France; 4 Roquette America Inc., 2211 Innovation Drive, Geneva, IL 60134, USA

**Keywords:** hydroxypropyl methylcellulose, mannitol, co-processed, aspirin, stability

## Abstract

Background/Objectives: Hydroxypropyl methylcellulose (HPMC) is one of the most commonly used hydrophilic polymers in formulations of matrix tablets for controlled release applications. However, HPMC attracts moisture and poses issues with drug stability in formulations containing moisture-sensitive drugs. Methods: Herein, the moisture sorption behavior of excipients and drug stability using aspirin as the model drug in matrix tablets were evaluated, using HPMC and the newly developed mannitol-coated HPMC, under accelerated stability conditions (40 °C, 75% relative humidity) with open and closed dishes. Results: Tablets prepared with mannitol-coated HPMC showed a slower drug degradation rate compared to tablets prepared with directly compressible HPMC. Initial moisture content and hygroscopicity were stronger predictors of drug stability compared to water activity when comparing samples without similar moisture content. In the early stage (day 0 to 30), the aspirin degradation rate was similar in both open and closed conditions, as moisture content is the main degradation contributor. In the later stage (day 30 to 90), aspirin degradation was faster under closed conditions than under open conditions, likely due to autocatalytic effects caused by the volatile acidic by-product entrapped in the closed environment. Conclusions: The findings from this study reinforced the importance of judicious excipient selection based on the understanding of excipient–moisture interactions to maximize the chemical stability of moisture-sensitive drugs. Mannitol-coated HPMC is a promising addition to the formulator’s toolbox for the formulation of controlled release dosage forms by direct compression.

## 1. Introduction

Hydroxypropyl methylcellulose (HPMC) is one of the most commonly used hydrophilic polymers in formulations of matrix tablets for controlled release applications [1]. Despite its ubiquity, issues with poor flowability render HPMC challenging to use for direct compression [2,3]. The attraction of employing direct compression for producing HPMC matrices stems not only from using an efficient and cost-effective manufacturing process but also from avoiding wet granulation of a highly viscous polymer. In addition, direct compression is ideal for heat or moisture-sensitive drugs [4,5]. Several companies developed direct compression-grade HPMC, such as METHOCEL™ DC2 (Colorcon) [6], Benecel™ DC (silicified HPMC, Ashland) [7], and RetaLac^®^ (co-processed HPMC–lactose, Meggle) [8], which were shown to deliver improved flow and compression attributes. However, the impact of hygroscopic HPMC when formulated with moisture-sensitive drugs is not well understood. It is generally perceived that a material that absorbs more than 5% moisture at relative humidities below 60% is considered hygroscopic [9].

A drug product should be stable in terms of its chemical, physical, and microbiological properties during its shelf life [10]. Chemical instability, in particular, is characterized by the reduction in labeled drug content and the presence of degradants. Drug degradation typically occurs by hydrolysis, oxidation, photolysis, or photodegradation [11]. Of these, hydrolysis of esters, amides, and carbamates is a major cause of degradation among active pharmaceutical ingredients [12]. Drug hydrolysis in solid dosage form occurs in the presence of moisture, from the initial moisture content of individual components used, or moisture from the external environment [11]. Drug degradation may compromise the safety and efficacy of drug products. In general, drug content should be within 5% *w*/*w* of its initial value and the presence of certain degradation products should be within pre-determined limits during its shelf-life [13].

In a finished product, drug stability can be maintained by the avoidance of adventitious environmental moisture through the use of moisture barriers in the form of product packaging [14], tablet coatings [15] and the inclusion of intra-package desiccants [12]. However, such mitigation strategies often incur additional costs. It is therefore desirable to employ a more cost-effective approach through formulation adjustments. For example, the use of starch as an intra-tablet desiccant that preferentially absorbs moisture has been proposed due to the presence of numerous hydroxyl groups coupled with open conformation that permit water entry [16]. This restricts the mobility of water molecules for hydrolysis reactions, thereby enhancing drug stability [17]. It is also notable that moisture in excipients may be transferred to drug particles when relative humidity (RH) in the microenvironment of the dosage form is altered, or by adsorbed moisture at the boundaries between excipient and drug particles [18]. Hence, it would be best to limit the formulation’s initial moisture content, which could be achieved by designing co-processed materials with low hygroscopicity while maintaining desired functionalities. For example, an anti-hygroscopic effect was demonstrated through particle surface coverage by crystalline L-leucine, which imparted resistance to the negative impact of moisture on aerosolization performance [19]. It was separately found that 96.5% of the particle surface could be shielded when using only 50% *w*/*w* of the hydrophobic material for effective moisture protection [20]. A similar approach was also reported whereby a coating of polyethylene glycol was applied to reduce the hygroscopicity of sodium carbonate, and it significantly improved flowability and processability [21].

Mannitol has one of the lowest hygroscopicities among commonly used tablet fillers [22]. It is a naturally occurring six-carbon sugar alcohol and is produced commercially by hydrogenation of fructose [23,24], most commonly derived from maize, wheat, or tapioca starches [25]. It is widely used in pharmaceutical formulations due to its chemical inertness, good physiological compatibility, and high compactability [22,26]. Micronized crospovidone co-grounded with mannitol had reduced hygroscopicity while maintaining good tablet physical stability [27]. Mannitol has also been added to poly(vinyl alcohol) tablet coatings to impart moisture-protective properties [28]. It is therefore interesting to explore the use of mannitol-coated HPMC particles as a moisture barrier to enable the formulation of moisture-sensitive drugs for controlled release.

In a previous study, co-processed HPMC–mannitol produced by spray coating mannitol over HPMC was shown to exhibit improved flowability, good tabletability, and maintained flexibility to obtain desired release profiles [29]. Despite the widespread use of HPMC, its impact on the stability of moisture-sensitive drugs has not been investigated. Furthermore, any change in excipients should be carefully evaluated with adequately designed stability studies. Aspirin is selected as the model for moisture-sensitive drugs due to its susceptibility to hydrolysis, which can be used to elucidate the impact of moisture on chemical degradation. This study aims to understand the effects of mannitol-coating on the moisture sorption properties of HPMC and the impact on the stability of HPMC-based tablets. Both open and closed dish conditions were used to evaluate drug stability under accelerated stability conditions (40 °C, 75% RH), with open conditions to mimic bottle packaging and closed conditions to mimic individually packed blisters.

## 2. Results

### 2.1. Particle Size Distribution of Neat and Co-Processed Excipients

**Figure 1:**
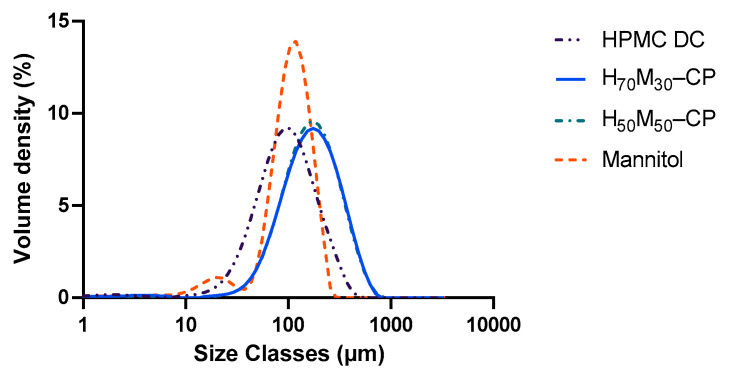
Particle size distribution of excipients studied.

The particle size distribution of the materials is shown in Figure 1, where the co-processed excipients showed slightly larger median particle sizes compared to the neat excipients. Spray-dried mannitol (D_50_ = 106.6 ± 1.2 µm; D_90_ = 176.6 ± 1.2 µm) of comparable size distribution to HPMC DC (D_50_ = 94.0 ± 1.4 µm; D_90_ 213.3 ± 9.4 µm) was selected for comparison in physical mixtures. The particle sizes of co-processed HPMC–mannitol (H_70_M_30_–CP: D_50_ = 165.8 ± 2.6 µm; D_90_ = 361.4 ± 5.2 µm and H_50_M_50_–CP: D_50_ = 166.7 ± 8.0 µm; D_90_ = 357.7 ± 13.2 µm) were larger than the HPMC DC particles.

### 2.2. The Relationship between Moisture Content and Water Activity

**Figure 2:**
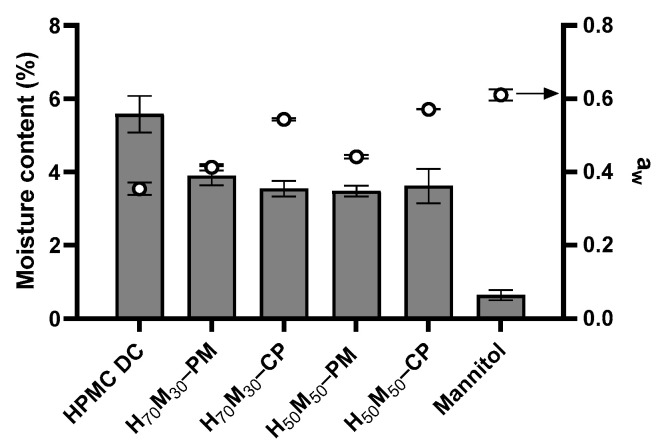
Moisture content and water activity of excipients studied. The bars represent moisture content while the points represent water activity. Error bars represent standard deviation (*n* = 3). There is no significant difference (*p* > 0.05) in moisture content between co-processed excipient and the corresponding physical mixture at equal ratios.

HPMC recorded a moisture content of 5.58 ± 0.50%, while non-hygroscopic mannitol recorded a moisture content of only 0.64 ± 0.14% (Figure 2). Moisture content decreased with increasing mannitol content in the physical mixtures but was similar between H_70_M_30_–CP and H_50_M_50_–CP. Despite having the lowest moisture content, the water activity of mannitol was the highest at 0.61 ± 0.02, indicating a greater tendency of this moisture to be free for reaction. In contrast, the water activity of HPMC was lower, at 0.35 ± 0.02. Surprisingly, co-processed HPMC–mannitol had similar moisture content but higher water activity than their corresponding physical mixtures at the same ratio (Figure 2). It is hypothesized that the mannitol coating slowed down moisture absorption into HPMC particles. During the time scale of the water activity measurements, less moisture is absorbed from the environment, resulting in higher water activity measured in co-processed excipients.

### 2.3. Moisture Sorption–Desorption Isotherm

**Figure 3:**
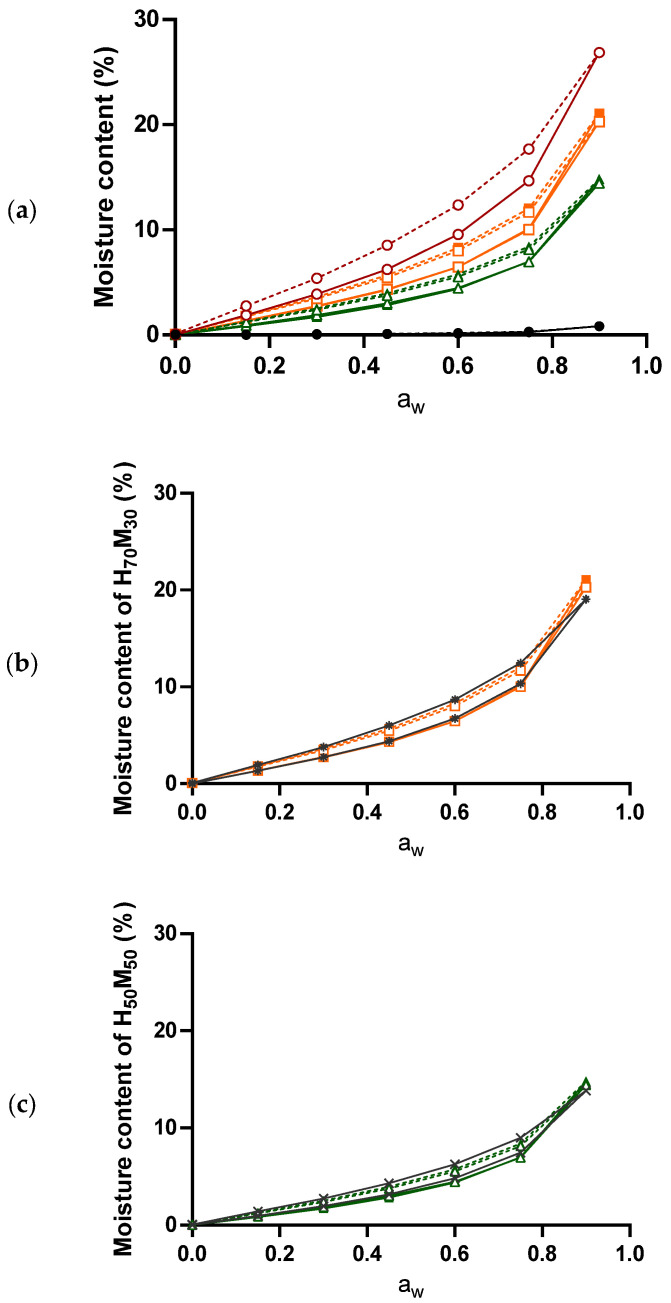
(**a**) Representative moisture sorption (solid lines) and desorption (dotted lines) isotherms of excipients studied, comparing (⚬) HPMC DC, (■) H_70_M_30_–CP, (□) H_70_M_30_–PM, (▲) H_50_M_50_–CP, (△) H_50_M_50_–PM and (●) mannitol. Comparison of experimental isotherm against theoretical weighted isotherm for (**b**) H_70_M_30_ and (**c**) H_50_M_50_; (*) theoretical weighted H_70_M_30_ and (×) theoretical weighted H_50_M_50_.

In Figure 3a, both HPMC and mannitol exhibited a Type II sorption isotherm [30], indicative of monolayer sorption by non-porous materials accompanied by subsequent multilayer sorption. Such physical characteristics of the studied materials have been similarly reported in previous studies [31,32]. Mannitol had negligible moisture sorption (<0.25%) up to 0.75 a_w_. At 0.90 a_w_, the moisture content of mannitol was only 0.83 ± 0.01%, indicative of a non-hygroscopic material. In contrast, HPMC had more significant moisture sorption even at 0.15 a_w_ (1.90 ± 0.00%), which increased with increasing water activity. Sorption isotherms of physical mixtures and co-processed excipients were similar to the weighted average of sorbed moisture by individual components at each water activity, as shown in Figure 3b,c. Such moisture sorption behavior is similar to the findings of Zhang and Zografi [33], using sugar and poly(vinyl pyrrolidone) mixtures.

**Figure 4:**
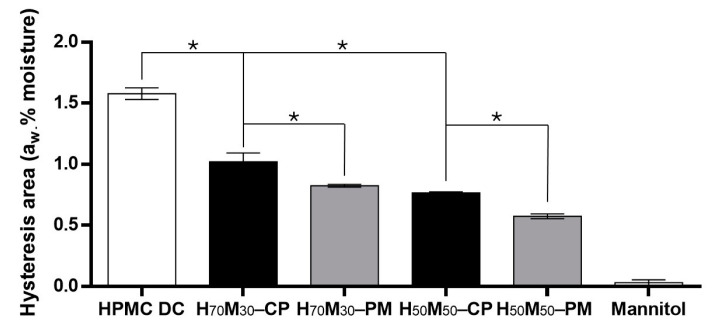
Area of hysteresis comparison between co-processed excipients and physical mixtures. Error bars represent standard deviation (*n* = 2). Asterisks indicate statistical significance (*p* < 0.05).

The hysteresis loop, represented by the area difference between the sorption and desorption isotherms, was greater in HPMC than in mannitol (Figure 4). Interestingly, higher hysteresis area was observed in co-processed excipients compared to physical mixtures at the same HPMC–mannitol ratio, where the hysteresis areas of H_70_M_30_–CP, H_70_M_30_–PM, H_50_M_50_–CP, and H_50_M_50_–PM were 0.98 ± 0.02, 0.82 ± 0.01, 0.77 ± 0.01 and 0.57 ± 0.02 a_w_·% moisture, respectively. This is hypothesized to be attributed to greater moisture entrapment by the mannitol coating, hence increasing the driving force for removing entrapped moisture.

### 2.4. Aspirin Degradation under Accelerated Stability Conditions

**Figure 5:**
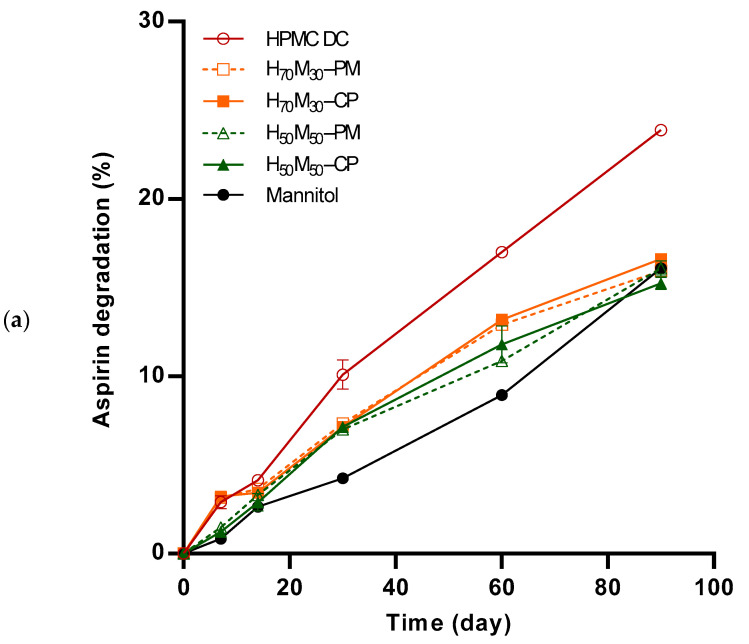

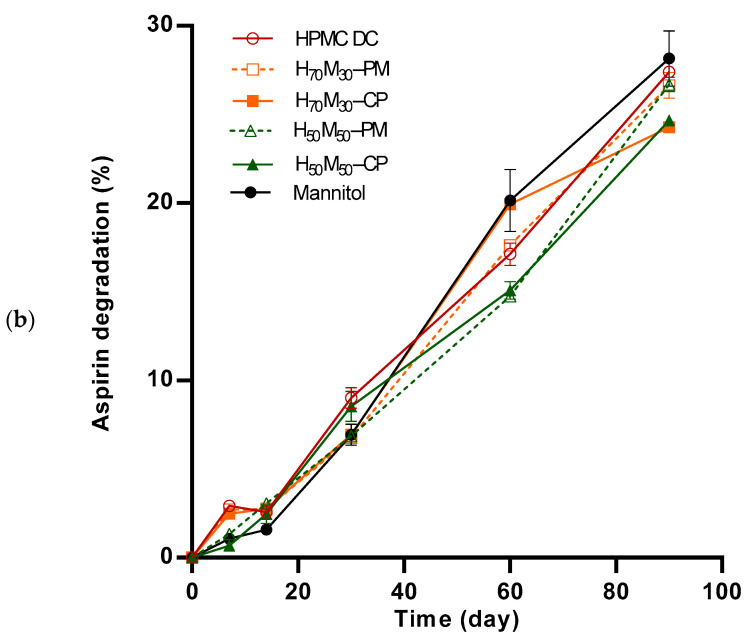
Aspirin degradation profiles during accelerated stability study, under (**a**) open and (**b**) closed conditions, at 40 °C and 75% RH. HPMC–mannitol ratio was varied: (⚬) HPMC DC, (■) H_70_M_30_–CP, (□) H_70_M_30_–PM, (▲) H_50_M_50_–CP, (△) H_50_M_50_–PM and (●) mannitol. Error bars represent standard deviation (*n* = 3).

In general, a slower degradation rate was observed in tablets containing more mannitol when the HPMC–mannitol ratio was decreased (Figure 5). HPMC DC-based tablets degraded the fastest under open conditions, recording a degradation of 23.9 ± 0.2% at the end of the 90-day study. In contrast, mannitol-based tablets degraded by only 16.1 ± 0.3% within the same duration under open conditions. Interestingly, samples under closed conditions saw faster degradation compared to samples under open conditions. Under closed conditions, mannitol-based tablets degraded the fastest, recording a degradation of 28.2 ± 1.6%. This is likely attributed to mannitol’s relatively lower affinity to moisture compared to HPMC. In the tablets, HPMC competes with drug particles for available moisture, preferentially absorbing it, thereby reducing the moisture accessible to the drug particle surfaces for degradation. Veronica et al. [34] similarly observed this phenomenon, wherein the inclusion of water-soluble fine salt crystals competed with drug particles for moisture, thereby mitigating the degradation of aspirin in comparison to tablets formulated without the salts.

**Figure 6:**
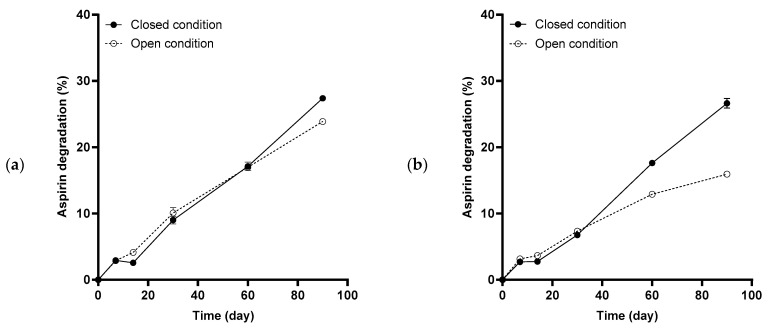

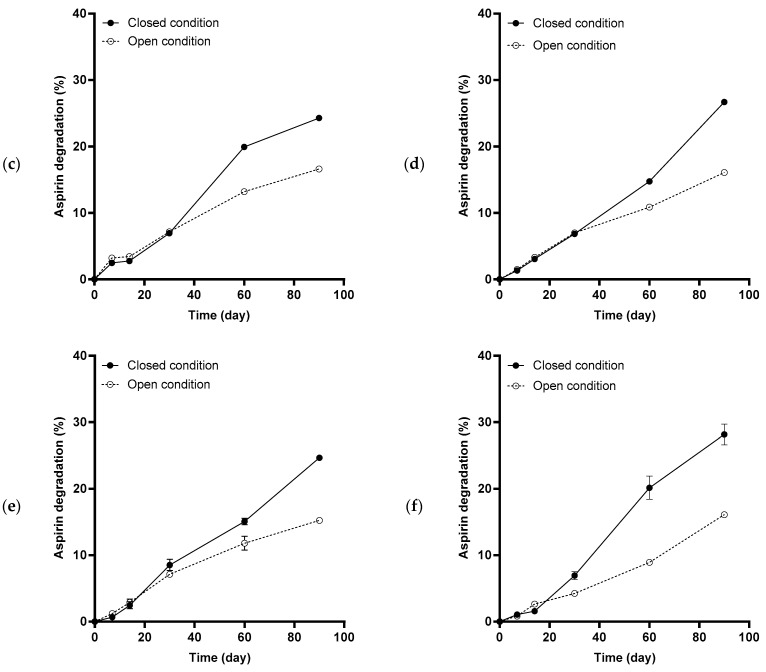
Aspirin degradation profiles during accelerated stability study at 40 °C and 75% RH, comparing samples exposed to closed (solid line) against open (dotted line) stability conditions. HPMC–mannitol ratio was varied: (**a**) HPMC DC, (**b**) H_70_M_30_–PM, (**c**) H_70_M_30_–CP, (**d**) H_50_M_50_–PM, (**e**) H_50_M_50_–CP and (**f**) mannitol. Error bars represent standard deviation (*n* = 3).

A comparison of the degradation profiles between open and closed conditions for the excipients and blends studied is presented in Figure 6. The early-stage degradation rate was similar between open and closed conditions, but two distinct slopes were observed during the later stage, where samples under closed conditions degraded faster than those under open conditions.

### 2.5. Aspirin Degradation Rate

Data from days 0 to 30 were fitted using the Leeson–Mattocks model representing degradation in the solid state and the degradation rates are presented in Table 1. The aspirin degradation data saw a good fit to the Leeson–Mattocks equation, with *R*^2^ > 0.95 for all fitted data. In general, open conditions saw a faster degradation rate than closed conditions. Under open conditions, a slightly slower degradation rate was observed in co-processed excipients compared to their corresponding physical mixtures (i.e., H_70_M_30_–CP vs. H_70_M_30_–PM: 5.10 vs. 5.22 × 10^−3^ day^−1^ and H_50_M_50_–CP vs. H_50_M_50_–PM: 4.70 vs. 4.79 × 10^−3^ day^−1^). However, this trend was reversed for samples stored under closed conditions, where a faster degradation rate was observed in co-processed excipients compared to physical mixtures (Table 1).

The factors affecting the degradation rate were investigated using Pearson’s product-moment correlation and the results are presented in Table 2. The increase in mannitol content reduced the aspirin degradation rate in tablets stored under both open (r = −0.872) and closed conditions (r = −0.943). Since mannitol content directly affected moisture content in the co-processed excipients and their corresponding physical blends, a positive correlation was found between degradation rates and moisture descriptors such as moisture content, sorption AUC, desorption AUC, and hysteresis area, in both open and closed conditions. At the same time, water activity had a strong negative correlation with open conditions (r = −0.837) and closed conditions (r = −0.943) degradation rates.

## 3. Discussion

It would be prudent to carefully risk assess the impact of substituting excipients on drug product performance and stability. This requires a thorough understanding of excipient–moisture interactions to identify possible failure modes and mitigation strategies. Herein, the extent of aspirin degradation in HPMC-based tablets, including the novel mannitol-coated HPMC, was evaluated under both open and closed accelerated stability conditions, to mimic tablets in bottles and individual blister packs, respectively. The extent of drug degradation differed with different excipients used and storage conditions.

### 3.1. Mannitol as a Moisture Protective Coating to Enhance Formulation Stability

Degradation of aspirin in the solid state occurs when aspirin dissolves in the water surrounding it and undergoes hydrolysis. The extent of degradation would depend on the amount of moisture present in the microenvironment, which affects the amount of moisture that could adsorb onto the aspirin particle surface. The source of moisture could either be from the environment in open stability conditions (dependent on the equilibrium RH) or sorbed moisture in neighboring excipients in the formulation in closed stability conditions. In the absence of moisture, aspirin decomposition is negligible [35]. The open conditions provide an environment whereby the degradation of moisture-sensitive aspirin occurs in a larger space, albeit within a controlled humidity environment, and is useful for understanding the moisture-dependent degradation of a bottled formulated product. A general consensus is that formulations containing hygroscopic excipients such as HPMC may accelerate drug degradation when microenvironment humidity is abundant [36]. Indeed, it was observed that tablets with HPMC DC degraded the fastest (Figure 5) while tablets formulated with mannitol showed the least degradation under open conditions. This has been similarly reported by Patel et al. [37], where higher aspirin degradation was observed when microcrystalline cellulose was added to the formulation. It was proposed that microcrystalline cellulose, by absorbing moisture from the environment, increased the likelihood of drug–water interactions and consequently led to more drug degradation through hydrolysis. This observation can be rationalized based on the understanding of moisture interaction with the excipients. Mannitol, a crystalline material, has low moisture uptake regardless of changes in RH conditions, while HPMC, a hydrophilic and hygroscopic polymer, possesses the capacity for significant moisture uptake even at low water activity conditions. In view of mannitol’s inertness towards moisture, mannitol-coated HPMC was strategically developed to impart stability-enhancing benefits.

Herein, tablets with H_70_M_30_–CP and H_50_M_50_–CP demonstrated improved chemical stability compared to tablets with HPMC DC under open conditions. By adjusting the ratio between HPMC and mannitol, less degradation was observed in tablets with more mannitol due to lower moisture sorption (Figure 2). In fact, a strong positive correlation between degradation rate and sorption AUC was observed (Table 2). More importantly, H_70_M_30_–CP and H_50_M_50_–CP saw slightly slower degradation rates compared to their corresponding physical mixtures under open conditions (Table 1). This could be attributed to the slightly lower moisture uptake of the co-processed excipients (Figure 3a), as mannitol coating reduced the available HPMC surface for water uptake from the environment. It is further postulated that the mannitol coating might have slowed down the swelling of HPMC particles in the solid state [38] and water absorption into the bulk [39], which reduced the moisture retentive capacity of HPMC. This was supported by the lower thickness of tablets containing H_70_M_30_–CP and H_50_M_50_–CP than those containing H_70_M_30_–PM and H_50_M_50_–PM, respectively, at later time points. Additionally, the hysteresis areas of H_70_M_30_–CP and H_50_M_50_–CP were larger than their respective physical mixtures (Figure 4), indicative of greater moisture entrapment due to the mannitol coating which could reduce the release of in situ water to degrade neighboring drug particles. Similarly, in another study, lower aspirin degradation was observed in tablets containing starches with greater hysteresis. This was attributed to the greater moisture-binding capacity of starch where water molecules preferentially interacted with starch binding sites rather than aspirin particles [17]. In contrast, the slightly higher degradation rates of tablets containing co-processed excipients than physical mixtures under closed conditions can be attributed to the higher initial moisture content of H_50_M_50_–CP compared to H_50_M_50_–PM (Figure 2), since the initial moisture content in excipients would play a more significant role when moisture from the microenvironment is limited.

These findings highlighted the benefits of mannitol-coated HPMC over HPMC DC by (a) limiting access to moisture thereby reducing the sensitivity of co-processed HPMC–mannitol to moisture uptake, and (b) reducing the tendency for the co-processed excipient to release moisture, as demonstrated by the larger hysteresis area compared to physical mixtures. However, the decrease in drug degradation rate when comparing tablets containing co-processed HPMC–mannitol or physical mixtures at identical ratios was not particularly pronounced. This could be attributed to fragmentation of the mannitol coating in the co-processed HPMC–mannitol excipient during tablet compression, since mannitol is known to undergo fragmentation during tablet compaction [40]. As a result, post-compaction, the spatial distribution of HPMC and mannitol within the tablets formulated with the co-processed HPMC–mannitol was not markedly different from the tablets formulated with physical mixtures. Nonetheless, the lower hygroscopicity of co-processed HPMC–mannitol can translate into improved ease of raw material management across storage, handling, and confirmatory analytical testing during the product lifecycle. Therefore, mannitol-coated HPMC can be used to mitigate moisture-induced drug stability issues if a hygroscopic excipient such as HPMC is required for drug release modification.

### 3.2. Relationship between Water Activity and Drug Stability

Under open conditions, a positive correlation between degradation rate and initial moisture content was observed (Table 2). In contrast, a negative correlation between degradation rate and water activity was observed. Water activity represents the ratio of vapor pressure above a sample over the vapor pressure of water at equilibrium [12]. In an excipient, the total water content may be present in the free form or bound due to physical interactions with hydrophilic functional groups in the excipient. Water activity describes the tendency for water molecules to be freed and has been used as an indication of free water that participates in hydrolysis [12]. Generally, high water activity indicates greater availability of free water for degradation reactions which may result in higher drug degradation rates [41,42]. With this understanding, the negative correlation (r = −0.837) between degradation rate and water activity may seem to be counterintuitive. This is postulated to be due to the vastly different hygroscopicity of excipients used—although the water activity of mannitol was the highest among excipients studied, its low affinity for water resulted in the lowest initial moisture content (0.60 ± 0.17%) and also the lowest amount of sorbed moisture across the range of water activities. The amount of water available for degradation is therefore inferred to be the lowest in absolute terms. The converse may also be true for HPMC. Hence, when comparing materials of different initial moisture contents, the impact of water activity was overshadowed by the primary effect of moisture content on the amount of free water available for reaction. In such cases, initial moisture content and hygroscopicity are thought to be the more appropriate predictors for drug degradation. Nevertheless, water activity may still be used to describe the tendency for drug degradation if samples compared are of similar moisture contents, such as when comparing starches from different botanical origins or between hygroscopic salts [17,43,44,45]. For example, tablets produced with wheat starch recorded a lower aspirin degradation rate compared to rice starch, probably due to its lower water activity [17]. Similarly, the degradation of aspartame in agar–microcrystalline cellulose gels decreased when water activity was reduced from 0.8 to 0.3 [46]. However, when comparing high water activity-low moisture content dicalcium phosphate against low water activity-high moisture content starch samples, it may not be fair to attribute aspirin degradation to water activity alone [47]. In fact, it was found that drug stability could not be fully described with water activity alone, whereby maize starch with the lowest water activity did not record the lowest aspirin degradation [17]. Therefore, the use of water activity as a predictor of drug stability should be endorsed with caution as it needs to be interpreted together with the initial moisture content of the sample.

### 3.3. Entrapment of Volatile Acidic Degradation By-Products Autocatalyzed Aspirin Degradation

Degradation under closed conditions would mimic how tablets behave in the blister packaging where moisture in the microenvironment is more limited. In this study, during the early stage of degradation, the degradation rates of samples under closed conditions were slightly lower than those under open conditions (Table 1). This has been similarly reported by Patel et al. [37]. This could be because the glass vials with limited moisture permeability protected the tablets from exposure to external moisture, hence tablets under closed conditions were less susceptible to degradation [12,48]. As the study progressed, the aspirin degradation rate became slower in both open and closed conditions, probably due to the increasingly difficult accessibility of moisture to aspirin particles that were further in the core of the tablet.

Interestingly, the trend in degradation rate was reversed in the later stage of the stability study between samples stored under open and closed conditions, where samples under closed conditions degraded faster than those under open conditions (Figure 6). This was surprising as slower hydrolysis reactions would generally be expected for closed conditions since the amount of moisture within the packaged product would be more limited and any degradation is generally attributed to initial moisture content and the resultant RH in the packaged product. This counterintuitive phenomenon could be explained by understanding the mechanism of aspirin autocatalytic degradation: elevated degradative reaction accelerated by elevated temperatures and the presence of alkali [49,50] or acidic by-products such as salicylic acid and acetic acid formed from the hydrolysis of the carboxyl ester bond during aspirin degradation [35,51]. In this study, under closed conditions, the formation of acetic acid vapor through aspirin degradation may have increased the internal vapor pressure within the enclosed environment, which might have further hampered the vaporization of the acetic acid by-product resulting in a liquid state of acetic acid being formed and trapped within the tablets. As a result, a higher concentration of acid remained around aspirin particles to catalyze further aspirin degradation. This mechanism has also been proposed by Siegel et al. [52] to explain the observation where moisture-sensitive drugs such as aspirin and ascorbic acid were more stable in more permeable containers during stability studies as the catalytic by-products could escape to the exterior environment. In another study, Patel et al. [37] attributed the faster degradation of aspirin–dicalcium phosphate tablets under closed conditions compared to open conditions to moisture liberation from excipients. This might not be the case in this study as the initial degradation rate was faster under open conditions than closed conditions, probably due to the greater access to moisture for reaction under open conditions. Autocatalysis due to entrapment of the volatile acetic acid degradation by-product may better describe the hastened drug degradation in the later stage, under the enclosed environment. Due to the snow-balling effect of autocatalysis on aspirin degradation, the impact of the HPMC–mannitol ratio on aspirin degradation was less obvious under closed conditions as a result. This finding highlighted the importance of conducting stability studies in the final packaging condition to account for auxiliary factors apart from temperature and humidity effects, and better estimate the actual shelf-life of the drug product. Nonetheless, mannitol-coated HPMC-based tablets improved drug stability when compared to HPMC DC-based tablets, as seen in the lower degradation rates in both open and closed conditions.

## 4. Materials and Methods

### 4.1. Materials

Direct compression grade HPMC (HPMC DC; METHOCEL™ DC2 K4M, HPMC 2208, 4000 mPa.s grade, Colorcon, Harleysville, PA, USA) was used as received. Co-processed HPMC–mannitol, prepared at a 70:30 ratio (H_70_M_30_–CP) and 50:50 ratio (H_50_M_50_–CP) were kind gifts from Roquette, France. These co-processed excipients were prepared using a spray-drying tower, where mannitol syrup was sprayed onto fluidized HPMC particles (Benecel™ K4M CR, Ashland, Wilmington, DE, USA) and simultaneously dried. As references for comparison, physical mixtures of HPMC DC and spray-dried mannitol (PEARLITOL^®^ 100 SD, Roquette, Lestrem, France) at different ratios were prepared. The ratio of HPMC and mannitol was varied at 70:30 and 50:50 to obtain H_70_M_30_–PM and H_50_M_50_–PM physical mixtures, respectively. Physical mixtures were prepared by blending HPMC and mannitol at the respective ratios in a tumble blender (Turbula^®^, WAB, Basel, Switzerland) at 42 rpm for 10 min. Aspirin (Euro Chemo-Pharma, Perai, Malaysia) was used as the model moisture-sensitive drug as its degradation products and mechanism of degradation in the solid state have been well characterized [35,50,53]. Magnesium stearate (MgSt; Productos Metalest, Zaragoza, Spain) was the tableting lubricant.

Lithium chloride and sodium chloride solutions (Meter Group, Pullman, WA, USA) were used for the calibration of the water activity meter (Aqualab 4TEV, Meter Group, Pullman, WA, USA). Salicylic acid (Sigma-Aldrich, Burlington, MA, USA) was used to prepare calibration standards for the quantification of aspirin degradation products by high-performance liquid chromatography (HPLC). Acetonitrile (J.T. Baker, Phillipsburg, NJ, USA), ortho-phosphoric acid (Sigma-Aldrich, Buchs, Switzerland), and purified water (Advantage A10, MilliQ, Burlington, MA, USA) were used to prepare the mobile phase for HPLC analyses. Dichloromethane (Merck, Darmstadt, Germany) and isopropyl alcohol (Avantor Performance Materials, Alberta, Canada) were used for particle sizing by laser diffractometry.

### 4.2. Particle Size Analysis

Laser diffractometry (Mastersizer 3000 Hydro MV, Malvern, Malvern, UK) was used to determine the particle size distribution of the powder samples. Dichloromethane was used to suspend HPMC–mannitol and mannitol while isopropyl alcohol was used to suspend pure HPMC. D_10_, D_50_, and D_90_ values were determined, which represented particle sizes at the 50th, and 90th volume percentiles under the cumulative undersize curve, respectively.

### 4.3. Determination of Moisture Content

In this study, moisture content was estimated using loss on drying. Briefly, weighed samples of approximately 1 g were evenly spread onto an aluminum pan and dried at 105 °C using a moisture analyzer (MB45, Ohaus, Parsippany, NJ, USA) for 15 min. The final sample weight was recorded as the dried weight. The moisture content of the sample was calculated using Equation (1). Three replicated runs were conducted, and the results were averaged.

(1) $$\mathrm{Moisture}\;\mathrm{content}\;(\%)=\frac{[\mathrm{Initial}\;\mathrm{weight}]\;-\;[\mathrm{Dried}\;\mathrm{weight}]\;}{[\mathrm{Initial}\;\mathrm{weight}]}\times 100$$

### 4.4. Determination of Water Activity

Water activity (a_w_) is the ratio of the vapor pressure above the sample to the vapor pressure of pure water. The water activity of approximately 1 g sample was determined using a dew point water activity meter (Aqualab 4TEV, Meter Group, Pullman, WA, USA) at 25 °C. The water activity meter was calibrated at 25 °C using 13.41 mol/kg lithium chloride solution for 0.25 ± 0.003 a_w_, 8.57 mol/kg lithium chloride solution for 0.50 ± 0.003 a_w_, and 6 mol/kg sodium chloride solution for 0.76 ± 0.003 a_w_ before measurement. Three replicated runs were conducted, and the results were averaged.

### 4.5. Generation of Moisture Sorption Isotherm

The container with the powder sample was tumbled five times before the sample was withdrawn. A sample of approximately 7 mg was loaded onto a metallized quartz pan and subjected to various temperature and humidity conditions in a dynamic vapor sorption analyzer (DVS; Q5000, TA Instruments, New Castle, DE, USA). Prior to sorption analysis, samples were first equilibrated at 60 °C and 0% RH for 60 min to remove initial moisture history. Moisture sorption isotherms were subsequently generated at 25 °C as water activity was stepped from 0 to 0.90 a_w_. Each experiment was conducted in duplicate. The areas under the curve (AUCs) for sorption (sorption AUC) and desorption isotherms (desorption AUC) were separately analyzed using GraphPad Prism (GraphPad Software, Version 9.0.1, Boston, MA, USA). The area of the hysteresis loop was determined as the difference between the sorption AUC and desorption AUC.

### 4.6. Preparation of Tablets

Blends containing aspirin (50% *w*/*w*) and excipient (49% *w*/*w*) were prepared by mixing at 42 rpm in a tumble blender (Turbula^®^, WAB, Basel, Switzerland) for 10 min followed by the addition of 1% *w*/*w* MgSt and mixing continued for another 2 min. For each blend, 400 mg tablets were prepared using a compaction simulator (STYL’One, Medelpharm, Beynost, France) equipped with Euro B 11.28 mm flat face punches (Kilian, Baden-Württemberg, Germany). The compression force was adjusted to achieve a target initial tablet tensile strength of approximately 1.2 MPa. The compression speed used was 40 mm/s.

### 4.7. Stability Studies

Stability studies were conducted as three independent groups across different days. Tablets were placed in stability chambers (SP Hotpack, SP Industries, Warminster, PA, USA) maintained at 40 °C and 75% RH, under open and closed conditions. For open-dish conditions, tablets were placed on Petri dishes, while for closed-dish conditions, tablets were placed in glass bottles with a lid. At pre-determined time points, tablets were withdrawn and assayed for aspirin and its degradation product, salicylic acid. Each assay was conducted in triplicates and the results were averaged. Five tablets were crushed, and three sets of 400 mg samples were separately withdrawn from the pulverized tablets.

**Figure 7:**
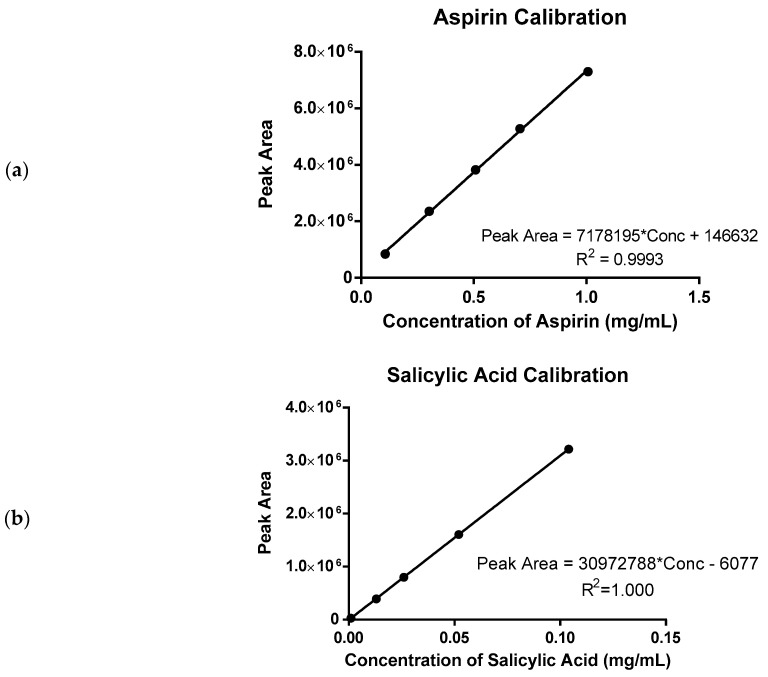

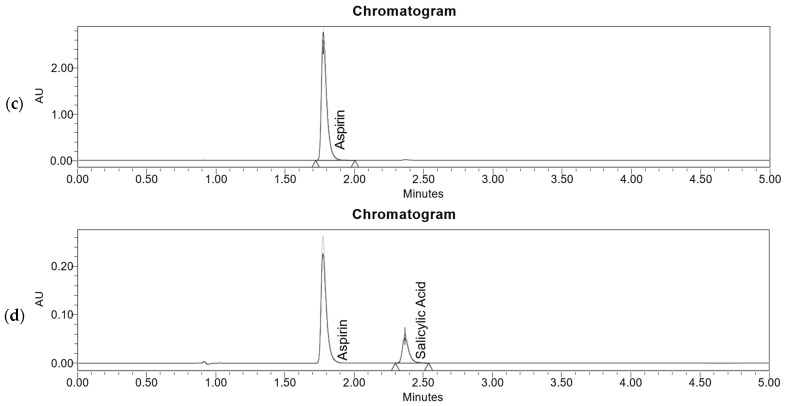
HPLC calibration curves for (**a**) aspirin and (**b**) salicylic acid and representative HPLC chromatograms at (**c**) 273 nm and (**d**) 296 nm.

The methods for sample preparation and assay were adapted from a previously reported method [54,55]. The sample was added to 25 mL of acetonitrile which functioned as extraction solvent. The suspension was sonicated for 1 min before centrifugation (Sorvall Primo R, Thermo Fisher Scientific, Waltham, MA, USA) at 4000 rpm for 5 min. An aliquot of 1.05 mL was taken from the supernatant, then added to 1.95 mL of purified water to obtain a total diluent ratio equivalent to the acetonitrile: aqueous phase ratio of 35:65 for HPLC analyses (1200 Series, Agilent, Santa Clara, CA, USA). Samples were subsequently diluted to the linear range of the calibration curve (Figure 7; up to 1.0 mg/mL for aspirin and up to 0.1 mg/mL for salicylic acid) for analysis using acetonitrile and purified water pre-mixed at the same ratio.

Samples were subsequently filtered with 0.45 μm regenerated cellulose filters (Sartorius, Göttingen, Germany) and 20 μL injected for HPLC analysis with a reversed-phase C18 column (Kinetex, 2.6 µm particle size, 4.6 mm × 100 mm, Phenomenex, Torrance, CA, USA) maintained at 40 °C. The mobile phase was acetonitrile and phosphate buffer (pH 1.65), 34.9:65.1 (*v*/*v*), and the flow rate was 0.9 mL/min. The detection wavelengths for aspirin and salicylic acid were 273 and 296 nm, respectively. The retention times for aspirin and salicylic acid were 1.8 and 2.4 min, respectively. Empower^®^ software (Waters, Version 3.6.0, Milford, MA, USA) was used for data analysis. The percentage of aspirin degradation was calculated using Equation (2).

(2) $$\mathrm{Aspirin}\;\mathrm{degradation}\;(\%)=\frac{\left[\mathrm{salicylic}\;\mathrm{acid}\right]}{\left[\mathrm{aspirin}\right]+\left[\mathrm{salicylic}\;\mathrm{acid}\right]}\times 100$$

where [salicylic acid] and [aspirin] represent the molar concentrations of salicylic acid and aspirin, respectively.

The aspirin degradation rate was estimated based on the Leeson–Mattocks equation (Equation (3)) [35] that described drug degradation in the solid state.

(3) $$\log\frac{A_{0}{\left({D_{0}}^{1/2}+C^{1/2}\right)}^{2}}{A{\left({D_{0}}^{1/2}+{C_{0}}^{1/2}\right)}^{2}}=\frac{{D_{0}}^{1/2}kp^{3n/2}t}{2.303}$$

*A*_0_ and *C*_0_ (in mole % × volume^−1^) are the initial aspirin and salicylic acid concentrations, respectively. *A* and *C* are the concentrations of aspirin and salicylic acid at time *t*, respectively. *D*_0_ refers to the total aspirin and salicylic acid concentration at any time point while *p*, *k,* and *n* refer to the vapor pressure, the degradation rate constant, and the order of the sorption reaction with respect to *p*, respectively.

### 4.8. Statistical Analyses

Graphical and statistical analyses were performed using GraphPad Prism (GraphPad Software, Version 9.0.1, Boston, MA, USA). Pearson’s correlation analysis was conducted to correlate the sample’s physical properties to the aspirin degradation rate. One-way analysis of variance (ANOVA) with post hoc Bonferroni’s multiple comparisons test was used to investigate the differences between the samples while the coefficient of determination (*R*^2^) represented the goodness of fit. The level of significance was defined at *p* < 0.05.

## 5. Conclusions

Judicious excipient selection based on the understanding of excipient-moisture interactions is important to maximize the chemical stability of moisture-sensitive drugs. The moisture protection effect of the non-hygroscopic mannitol coating in the controlled release HPMC–mannitol tablets was demonstrated, whereby tablets made with co-processed excipients (H_70_M_30_–CP and H_50_M_50_–CP) degraded at a slower rate compared to HPMC DC, although the difference in drug degradation rate when comparing tablets containing co-processed HPMC–mannitol or physical mixtures at identical ratio was not particularly pronounced. Initial moisture content and hygroscopicity were shown to be stronger predictors of drug stability compared to water activity when comparing samples with different initial moisture contents because drug degradation is dependent on the amount of free water available for reaction. The higher late-stage drug degradation rate observed under closed conditions compared to open conditions could be attributed to the autocatalytic effects of the entrapped volatile acidic by-product. Hence, it is important to control initial moisture content in excipients and dosage forms to reduce drug degradation and subsequent autocatalytic chain events. The advantage of mannitol-coated HPMC over HPMC alone in controlled release formulations was demonstrated through improved stability of a moisture-sensitive drug, in addition to improved flowability, good tabletability, and tunable release kinetics as discussed in an earlier study [29]. Mannitol-coated HPMC is a promising addition to the formulator’s toolbox for the formulation of controlled release dosage forms by direct compression.

## Figures and Tables

**Table 1:** Degradation rates of aspirin tablets when HPMC–mannitol ratio was varied, and when exposed to open and closed stability conditions. *R*^2^ indicates the fitting of aspirin degradation data to the Leeson–Mattocks equation.

Excipient	Storage Condition			
	Open		Closed	
	Degradation Rate (×10^−3^ day^−1^)	*R* ^2^	Degradation Rate (×10^−3^ day^−1^)	*R* ^2^
HPMC DC	6.36	0.99	5.58	0.97
H_70_M_30_–PM	5.22	0.95	4.72	0.97
H_70_M_30_–CP	5.10	0.95	4.76	0.96
H_50_M_50_–PM	4.79	1.00	4.65	1.00
H_50_M_50_–CP	4.70	1.00	5.08	0.98
Mannitol	3.33	0.98	4.26	0.98

**Table 2:** Pearson’s product-moment correlation coefficient for aspirin degradation rate with moisture descriptors.

Degradation Rate	Mannitol (%, *w*/*w*)	Moisture Content (%)	Sorption AUC	Desorption AUC	Hysteresis Area	a_w_
Open conditions	−0.872 ^a^	0.981 ^c^	0.990 ^c^	0.991 ^c^	0.973 ^b^	−0.837 ^a^
Closed conditions	−0.943 ^a^	0.892 ^a^	0.826 ^a^	0.844 ^a^	0.910 ^a^	NS

^a^ statistically significant, *p* < 0.05; ^b^ statistically significant, *p* < 0.005; ^c^ statistically significant, *p* < 0.001; NS represents no statistical significance.

## Data Availability

Data is contained within the article.
